# Scale-Free Spanning Trees and Their Application in Genomic Epidemiology

**DOI:** 10.1089/cmb.2020.0500

**Published:** 2021-10-13

**Authors:** Yury Orlovich, Kirill Kukharenko, Volker Kaibel, Pavel Skums

**Affiliations:** ^1^ Faculty of Applied Mathematics and Computer Science, Belarusian State University, Minsk, Belarus.; ^2^ Institute for Mathematical Optimization, Otto von Guericke University Magdeburg, Magdeburg, Germany.; ^3^ Department of Computer Science, Georgia State University, Atlanta, Georgia, USA.

**Keywords:** computational complexity, genomic epidemiology, integer linear programming, scale-free network, transmission network

## Abstract

**We study the algorithmic problem of finding the most “scale-free-like” spanning tree of a connected graph. This problem is motivated by the fundamental problem of genomic epidemiology: given viral genomes sampled from infected individuals, reconstruct the transmission network (“who infected whom”). We use two possible objective functions for this problem and introduce the corresponding algorithmic problems termed** *m***-SF (-scale free) and** *s***-SF Spanning Tree problems. We prove that those problems are APX- and NP-hard, respectively, even in the classes of cubic and bipartite graphs. We propose two integer linear programming (ILP) formulations for the** *s***-SF Spanning Tree problem, and experimentally assess its performance using simulated and experimental data. In particular, we demonstrate that the ILP-based approach allows for accurate reconstruction of transmission histories of several hepatitis C outbreaks.**

## 1. Introduction

Viral outbreaks continue to be major causes of morbidity and mortality. The ongoing pandemic of the coronavirus SARS-CoV-2 (Huang et al., 2020) is a vivid example, but long-standing epidemics of HIV, hepatitis B virus, and hepatitis C virus (HCV) are hardly less damaging (Kilmarx, 2009; Hajarizadeh et al., 2013). Viral epidemics are complex processes defined by evolutionary dynamics of pathogens and social dynamics of susceptible populations (e.g., individual behaviors, social interactions, and mobility patterns).

Recent advances in sequencing technologies invigorated the field of genomic epidemiology (Armstrong et al., 2019; Knyazev et al., 2020) that aims to use viral genomic data to understand the epidemiological dynamics of pathogens. The fundamental algorithmic problem of genomic epidemiology could be formulated as follows:

- Given viral genomes sampled from *n* infected individuals, infer a transmission network indicating who of them infected whom (Knyazev et al., 2020). If each individual is supposed to be infected only once, then a transmission network is a tree called a *transmission tree*.

This problem has been approached by a variety of methods (Jombart et al., 2011, 2014; Sledzieski et al., 2019; Wertheim et al., 2014; Campo et al., 2016; De Maio et al., 2016; Klinkenberg et al., 2017; Skums et al., 2018). One family of methods is based on the so-called network approach. It is particularly popular among researchers of HIV and HCV and has been adopted as a standard methodology for outbreak investigations carried out by the CDC (Wertheim et al., 2014; Campo et al., 2016; Campbell et al., 2017; Kosakovsky Pond et al., 2018; Ramachandran et al., 2018; Ragonnet-Cronin et al., 2019). This approach usually consists of two stages. First, a weighted *relatedness graph G_R_* is constructed. Its vertices represent infected hosts, and edges connect the hosts whose viral populations are close to each other according to a selected population genetics measure. Often *G_R_* itself supplies enough information for epidemiologists and provides a *fast and scalable alternative* to phylogenetic trees when applied to next-generation sequencing (NGS) data (Wertheim et al., 2014; Campo et al., 2016; Ragonnet-Cronin et al., 2019). However, usually it contains many edges that do not represent actual transmissions. Thus, at the second stage, the transmission tree is inferred as the spanning tree of *G_R_*.

Under the maximum parsimony criterion, the most likely transmission network is a minimum spanning tree of *G_R_* (Jombart et al., 2011). However, experiments demonstrated that this approach is not accurate (Jombart et al., 2014). Furthermore, genomic data alone often do not allow to resolve ambiguities in transmission tree inference, and incorporation of additional evidence is necessary (Jombart et al., 2014; Villandre et al., 2016; Jha et al., 2017). Such evidence usually comes in the form of epidemiological information, such as sample collection times and exposure intervals. However, HIV, HCV, and many other infections tend to be initially asymptomatic, and consequently, sampling times may not accurately reflect the infection times. In addition, in outbreaks with high transmission rates (e.g., HIV/HCV among injection drug users), susceptible hosts are almost constantly exposed to the virus, which makes exposure intervals useless. Another important drawback of many existing methods is their implicit assumption that transmission tree edges are independent. In reality, it is not the case, as, for example, certain hosts (so-called superspreaders) infect more people than an average person (Galvani and May, 2005).

Skums et al. (2018) proposed an alternative approach. It is known that for viruses, whose transmissions are associated with behavioral risk factors, their transmission trees have properties of so-called scale-free graphs (Leigh Brown et al., 2011; Wertheim et al., 2014). Those graphs have specific features, including power-law degree distribution, small diameter, and the presence of high-degree vertices (hubs). This observation gives rise to the following informally defined algorithmic problem (*scale-free spanning tree problem*): find the most “scale-free-like” spanning tree *T* of the graph *G_R_*. In addition, constraints on the weight of *T* could be imposed. This approach was the basis of the Bayesian framework and the Markov Chain Monte Carlo algorithm for the transmission network inference described by Skums et al. (2018) and implemented as a tool called QUENTIN. Although QUENTIN is efficient in practice, it is a heuristic, and the questions about computational complexity and possibility of the exact solution of the problem were left open.

In this article, we present the first detailed study of the scale-free spanning tree problem. Our major contributions are as follows.

(1) We propose two rigorous formulations of the scale-free spanning tree problem further referred to as *m*-SF Spanning Tree and *s*-SF Spanning Tree problems. They are based on two related objective functions and, to the best of our knowledge, have not been previously studied.
(2) We establish the computational complexity of both problems by demonstrating that they are NP-hard or APX-hard, even when restricted to cubic graphs and bipartite graphs.
(3) We propose two integer linear programming (ILP) formulations for the problems, and perform computational experiments to assess their performance using simulated data. Then we apply an ILP approach to real genomic data from several epidemiologically curated HCV outbreaks investigated by the CDC (Campo et al., 2016; Skums et al., 2018) and demonstrate that it allows for accurate inference of transmission trees.

## 2. Preliminaries

### 2.1. Problem formulations

We consider only finite undirected simple graphs and use standard graph-theoretic terminologies, see, for example, Chartrand et al. (2016). Let G=(V,E) be a connected graph. For a vertex x∈V(G), the *neighborhood* $N_{G}\left(x\right)$ of *x* is the set of all vertices that are adjacent to *x* in *G*. The *degree* of *x* is defined as $\deg_{G}x=|N_{G}\left(x\right)|$. Several definitions of scale-free graphs of different degrees of mathematical rigor are known in a literature. We utilize the rigorous combinatorial characterization that has been introduced by Li et al. (2005) using the so-called *s*-metric of a graph. This graph invariant is defined as follows:

(1) $$s\left(G\right)=\sum_{uv\in E\left(G\right)}\deg_{G}u\deg_{G}v.$$

The same parameter is known in mathematical chemistry as *second Zagreb index* (Das and Gutman, 2004; Borovicanin et al., 2017). Li et al. (2005) demonstrated that a higher *s*-metric indicates with high probability the presence of most of the expected properties of scale-free graphs. The intuition behind these results is that in a graph with a high *s*-metric, a large number of edges should be incident to high-degree vertices, thus forcing them to resemble preferential attachment graphs—a standard Barabási and Albert (1999) model for scale-free networks. Therefore, another mathematical chemistry parameter called the *first Zagreb index* (Borovicanin et al., 2017) or *m-metric* also can serve as a measure of “scale-freeness” of a graph:


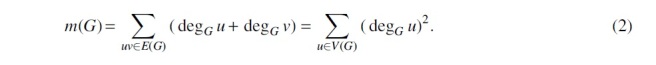


Thus, we can formulate *m*-SF Spanning Tree and *s*-SF Spanning Tree problems: given a connected graph *G*, find the spanning tree *T* of *G* such that m(T) (respectively, s(T)) is maximal. The respective maximum values of m(T) and s(T) are called *first* and *second SF-dimensions* of *G* and denoted by $\tau_{1}\left(G\right)$ and $\tau_{2}\left(G\right)$. By $T^{sopt}$ and $T^{mopt}$, we denote an *s-optimal tree* and an *m-optimal tree* of *G*, respectively.

A somehow related problem has been studied by Kincaid et al. (2016): find a spanning subgraph with *prescribed vertex degrees* such that its *s*-metric is maximum. This problem is polynomially solvable in general, but becomes NP-hard, when the output spanning subgraph is required to be connected.

### 2.2. Mathematical preliminaries

#### 2.2.1. Subgraph counting

Here we establish the characterizations for the *m*-metric and *s*-metric in terms of numbers of small subgraphs in a graph. This technique is used to establish complexity results in Section 3 and ILP formulations in Section 4.

**Proposition 1.** *For any graph G*,

$$m\left(G\right)=2\gamma_{2}\left(G\right)+2\gamma_{1}\left(G\right),s\left(G\right)=3\gamma_{\Delta }\left(G\right)+\gamma_{3}\left(G\right)+2\gamma_{2}\left(G\right)+\gamma_{1}\left(G\right),$$

*where* $\gamma_{\Delta }\left(G\right)$ *is the number of triangles and* $\gamma_{t}\left(G\right)$ *is the number of paths of length t in G*, *respectively.*

*Proof.* We prove only the second equality, the first one can be proved similarly. Let $A=\left[a_{ij}\right]$ be the adjacency matrix of *G* and ***d*** be its degree vector. We have $s\left(G\right)=\frac{1}{2}d^{T}\cdot A\cdot d$ and d=A⋅1, where 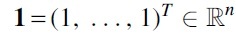. Therefore

$$s\left(G\right)=\frac{1}{2}1^{T}\cdot A^{3}\cdot 1=\frac{1}{2}\sum_{i=1}^{n}\sum_{j=1}^{n}a_{ij}^{\left(3\right)},$$

where 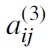 denotes 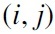-entry in the matrix *A*^3^.

It is known that $a_{ij}^{\left(3\right)}$ equals the number of walks of length 3 between vertices *i* and *j*. Thus, s(G) is equal to one-half of the total number of three-walks in *G*. An edge $v_{1}v_{2}$ produces exactly two such walks: $W_{11}=\left(v_{1},v_{2},v_{1},v_{2}\right)$ and $W_{12}=\left(v_{2},v_{1},v_{2},v_{1}\right)$. Each 2-path $\left\{v_{1}v_{2},v_{2}v_{3}\right\}$ produces four 3-walks: $W_{21}=\left(v_{1},v_{2},v_{3},v_{2}\right)$, $W_{22}=\left(v_{2},v_{3},v_{2},v_{1}\right)$, $W_{23}=\left(v_{2},v_{1},v_{2},v_{3}\right)$, and $W_{24}=\left(v_{3},v_{2},v_{1},v_{2}\right)$. Each 3-path $\left\{v_{1}v_{2},v_{2}v_{3},v_{3}v_{4}\right\}$ produces two 3-walks: $W_{31}=\left(v_{1},v_{2},v_{3},v_{4}\right)$ and $W_{32}=\left(v_{4},v_{3},v_{2},v_{1}\right)$. Finally, each triangle with vertex set $\left\{v_{1},v_{2},v_{3}\right\}$ produces six 3-walks: $W_{\Delta 1}=\left(v_{1},v_{2},v_{3},v_{1}\right)$, $W_{\Delta 2}=\left(v_{1},v_{3},v_{2},v_{1}\right)$, $W_{\Delta 3}=\left(v_{2},v_{3},v_{1},v_{2}\right)$, $W_{\Delta 4}=\left(v_{2},v_{1},v_{3},v_{2}\right)$, $W_{\Delta 5}=\left(v_{3},v_{1},v_{2},v_{3}\right)$, and $W_{\Delta 6}=\left(v_{3},v_{2},v_{1},v_{3}\right)$. As every three-walk of *G* has one of these forms, the statement of the lemma follows.□

#### 2.2.2. Neighbor switching

This is a tree rearrangement technique that is used for obtaining structural and complexity results. Let *T* be a tree and (u,v) be a pair of distinct vertices u,v∈V(T), where $\deg_{T}u=p\geq 2$ and $\deg_{T}v=t\geq 2$. We denote the unique u−v path in *T* by $P_{T}\left(u,v\right)$, and neighbors of *u* and *v* laying on $P_{T}\left(u,v\right)$ by $u^{+}$ and $v^{-}$, respectively. In case *u* and *v* are not adjacent, the neighbor of $u^{+}$ distinct from *u* and laying on $P_{T}\left(u,v\right)$ is denoted by $u^{++}$. Let $A=N_{T}\left(u\right)\setminus \left\{u^{+}\right\}=\left\{a_{1},\ldots ,a_{p-1}\right\}$, and let the set $N_{T}\left(v\right)\setminus \left\{v^{-}\right\}$ be partitioned into two subsets $B=\left\{b_{1},\ldots ,b_{q}\right\}$ and $C=\left\{c_{1},\ldots ,c_{r}\right\}$, where B≠. Furthermore, let $\deg_{T}u^{+}=\alpha$ and $\deg_{T}v^{-}=\beta$. Define numbers *D_A_*, *D_B_*, and *D_C_* as follows:

(3) DA=∑i=1p−1 degTai,DB=∑j=1q degTbj,DC=∑k=1r degTck.

**FIG. 1.**
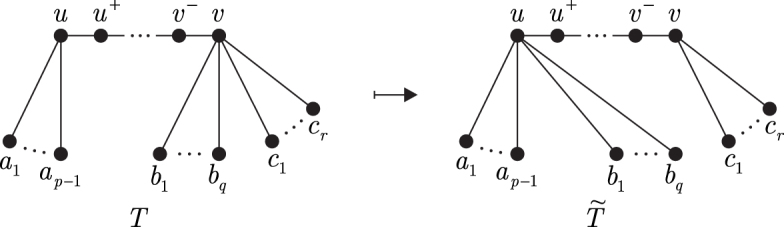
Neighbor switch.

Given the pair (u,v), the *neighbor switch* $S_{v\to u}^{B}$ is a transformation producing a new tree $\tilde{T}$ from *T* by replacing the edges $vb_{1},\ldots ,vb_{q}$ with new edges $ub_{1},\ldots ,ub_{q}$ (Fig. 1). This operation changes only degrees of the vertices *u* and *v*, namely $\deg_{\tilde{T}}u=p+q$, $\deg_{\tilde{T}}v=r+1$.

**Lemma 2.** *Suppose that* $S_{v\to u}^{B}\left(T\right)=\tilde{T}$*. If* p≥r+1*,* $D_{A}>D_{C}$ *and, in case u and v are not adjacent, additionally* α≥β*, then* $s\left(\tilde{T}\right)>s\left(T\right)$.

*Proof.* We prove lemma when *u* and *v* are not adjacent, that is, $u\neq v^{-}$ and $v\neq u^{+}$ (the proof for the other case is similar). Define by *X* (resp., *Y*) the set of edges of *T* (resp., $\tilde{T}$) incident to *u* or *v*. Let us denote by λ(X) (resp., $\tilde{\lambda }\left(Y\right)$) the contribution to s(T) (resp., $s\left(\tilde{T}\right)$) from the edges of *X* (resp., *Y*). Then

(4) $$s\left(\tilde{T}\right)-s\left(T\right)=\tilde{\lambda }\left(Y\right)-\lambda \left(X\right).$$

Using Equation (3) one can easily calculate

λ(X)=degTu degTu++degTv−degTv+∑i=1p−1 degTu degTai+∑j=1q degTv degTbj+∑k=1r degTvdegTck=pα+βt+pDA+tDB+tDC.

After substituting t=q+r+1, we obtain

(5) $$\lambda \left(X\right)=p\alpha +\beta q+\beta \left(r+1\right)+pD_{A}+qD_{B}+\left(r+1\right)D_{B}+qD_{C}+\left(r+1\right)D_{C}.$$

Similarly,

(6) $$\tilde{\lambda }\left(Y\right)=p\alpha +q\alpha +\beta \left(r+1\right)+pD_{A}+qD_{A}+pD_{B}+qD_{B}+\left(r+1\right)D_{C}.$$

Using equalities (4)–(6) we obtain

(7) $$\begin{array}{c} s\left(\tilde{T}\right)-s\left(T\right)=\tilde{\lambda }\left(Y\right)-\lambda \left(X\right)=q\alpha +qD_{A}+pD_{B}-\beta q-\left(r+1\right)D_{B}-qD_{C}\\ =q\left(\alpha -\beta \right)+D_{B}\left(p-r-1\right)+q\left(D_{A}-D_{C}\right). \end{array}$$

Since α≥β and p≥r+1, it follows that $q\left(\alpha -\beta \right)+D_{B}\left(p-r-1\right)\geq 0$. On the contrary, since q≥1 and $D_{A}>D_{C}$, we have $q\left(D_{A}-D_{C}\right)>0$ and therefore $s\left(\tilde{T}\right)-s\left(T\right)>0$.

If $B=N_{T}\left(v\right)\setminus \left\{v^{-}\right\}$, then the neighbor switch produces a tree $\tilde{T}$ with *v* being a leaf. In this case $S_{v\to u}^{B}$ is a *total neighbor switch*. For our goals it suffices to prove the following corollary.

**Corollary 3*.*** *If* $\tilde{T}$ *is obtained from T by a total neighbor switch* $S_{v\to u}^{B}$ *and, in case of u and v not being adjacent, additionally* α≥β *or* p≥β*, then* $s\left(\tilde{T}\right)>s\left(T\right)$.

*Proof.* We check that all conditions of Lemma 2 are satisfied for the total neighbor switch. Indeed, since $D_{A}\geq p-1\geq 1$ (recall $\deg_{T}u=p\geq 2$) and $D_{C}=r=0$, we have $D_{A}>D_{C}$ and p≥r+1. If *u* and *v* are not adjacent, we still require that α≥β, as in Lemma 2. However, this condition can be replaced if we rewrite Equation (7) as follows:

$$s\left(\tilde{T}\right)-s\left(T\right)=q\left(\alpha -\beta \right)+D_{B}\left(p-1\right)+qD_{A}=q\left(\alpha +D_{A}-\beta \right)+D_{B}\left(p-1\right).$$

Note that the latter expression is positive in case of p≥β, since α≥2 and $D_{A}\geq p-1\geq 1$.

In the same way we can compare the trees *T* and $\tilde{T}=S_{v\to u}^{B}\left(T\right)$ in terms of their *m*-metrics.

**Lemma 4.** *Suppose that* $S_{v\to u}^{B}\left(T\right)=\tilde{T}$ *and* p>r+1*. Then* $m\left(\tilde{T}\right)>m\left(T\right)$.

*Proof.* The idea is similar to the proof of Lemma 2. Since the neighbor switch changes only degrees of vertices *u* and *v*, $m\left(\tilde{T}\right)-m\left(T\right)=\deg_{\tilde{T}}^{2}u+\deg_{\tilde{T}}^{2}v-\deg_{T}^{2}u-\deg_{T}^{2}v=2q\left(p-r-1\right)$, which proves the lemma, since q≥1.

For further results we need weaker modifications of Lemmas 2 and 4 for the case $\deg_{T}u=p\geq 1$ (and therefore $D_{A}\geq 0$). Recall $\deg_{T}v=t\geq 2$ since we still require at least one vertex to switch.

**Lemma 5.** *Suppose* $\tilde{T}$ *is obtained from T by a total neighbor switch* $S_{v\to u}^{B}$*, then the following propositions hold:*

(*a)* $m\left(\tilde{T}\right)\geq m\left(T\right)$;
*(b)* $s\left(\tilde{T}\right)\geq s\left(T\right)$ *(unless u and v are not adjacent with* α<β*)*.

Now we consider a special case, when *u* is a vertex of maximum degree in *T* and all vertices in $N_{T}\left(v\right)\setminus \left\{v^{-}\right\}$ are leaves. In addition, let B,C≠. We introduce a *double neighbor switch* $S_{v\to u,u^{+}}^{B,C}\left(T\right)=S_{v\to u^{+}}^{C}\left(S_{v\to u}^{B}\left(T\right)\right)$. The reason to treat this two-step switch as a single operation is that the first switch itself might cause the descend of *s*-metric, however, the decrease would be compensated by the second switch.

**Lemma 6.** *If* $\hat{T}$ *is obtained from T by a double neighbor switch* $S_{v\to u,u^{+}}^{B,C}\left(T\right)$*, then* $s\left(\hat{T}\right)>s\left(T\right)$.

*Proof.* In case *u* and *v* are adjacent, that is, $u^{+}=v$, a double switch $S_{v\to u,u^{+}}^{B,C}\left(T\right)$ gets reduced to the first neighbor switch $S_{v\to u}^{B}\left(T\right)$, which produces a tree with a higher *s*-metric due to Lemma 2. Therefore, assume *u* and *v* are not adjacent. Consider the first switch and let $\tilde{T}=S_{v\to u}^{B}\left(T\right)$. From Equation (7), since $D_{B}=q$ and $D_{C}=r$, we obtain

(8) $$s\left(\tilde{T}\right)-s\left(T\right)=q\left(\alpha +D_{A}-\beta \right)+q\left(p-r-1\right)-qr.$$

Next let $\hat{T}=S_{v\to u^{+}}^{C}\left(\tilde{T}\right)$ be obtained by the total neighbor switch. To avoid reassigning of notations we denote $D_{E}=\sum_{w\in N_{\tilde{T}}\left(u^{+}\right)\setminus \left\{u^{++}\right\}}\deg_{\tilde{T}}w$ and $\gamma =\deg_{\tilde{T}}u^{++}$. Other notations stay the same from the first switch. Again from Equation (7) we get

(9) $$s\left(\hat{T}\right)-s\left(\tilde{T}\right)=r\left(\gamma -\beta \right)+r\left(\gamma -1\right)+rD_{E}.$$

Summation of Equations (8) and (9) gives

$$s\left(\hat{T}\right)-s\left(T\right)=q\left(\alpha +D_{A}-\beta \right)+q\left(p-r-1\right)+r\left(\gamma +D_{E}-\beta -q\right)+r\left(\gamma -1\right),$$

where $D_{A}\geq p-1$, α≥2, γ≥2, and $D_{E}\geq \deg_{\tilde{T}}u=p+q$. Furthermore, since *u* is a vertex of maximum degree in *T*, p≥β and p≥q+r+1>r+1 (recall q,r>0), which proves the lemma.

### 2.3. Bounds in terms of the maximum degree

There exist bounds for both SF-dimensions of a graph in terms of its order only (de Caen, 1998; Das, 2003; Das and Gutman, 2004). However, they are not particularly efficient, when used as ILP cuts. Here we provide the adjusted upper bounds that turned out to be more useful for that purpose. Let Δ(G) denote the maximum vertex degree of *G* and $S_{m,k}$ denote a *double star*, that is, a tree obtained from two disjoint stars $K_{1,m}$ and $K_{1,k}$ with *m* and *k* leaves, respectively, by adding an edge joining their central vertices.

**Theorem 7.** *For any graph G of order* n≥2*,*

$$\begin{array}{c} \tau_{1}\left(G\right)\leq m\left(S_{\Delta \left(G\right)-1,n-\Delta \left(G\right)-1}\right)=2\Delta^{2}\left(G\right)+n^{2}-2n\Delta \left(G\right)+n-2,\\ \tau_{2}\left(G\right)\leq s\left(S_{\Delta \left(G\right)-1,n-\Delta \left(G\right)-1}\right)=n\left(n-\Delta \left(G\right)-1\right)+\Delta^{2}\left(G\right). \end{array}$$

*Proof.* We provide the proof for the second SF-dimension only (the other proof is similar). Suppose $T^{sopt}$ is an *s*-optimal tree of *G* and $T^{sopt}\neq S_{\Delta \left(G\right)-1,n-\Delta \left(G\right)-1}$. We prove the statement by performing a sequence of neighbor switches on $T^{sopt}$, with each of them increasing *s*-metric, so that the resulting tree is $S_{\Delta \left(G\right)-1,n-\Delta \left(G\right)-1}$.

Let *u* be a vertex of maximum degree in $T^{sopt}$. Then for every *v* in $T^{sopt}$ follows $\deg_{T^{sopt}}v\leq \deg_{T^{sopt}}u\leq \deg_{G}u\leq \Delta \left(G\right)$. Let $T:=T^{sopt}$. We divide the sequence of neighbor switches into three stages.

Stage 1: *For each vertex v with all vertices in* $N_{T}\left(v\right)\setminus \left\{v^{-}\right\}$ *(where* $v^{-}\in P_{T}\left(u,v\right)$*) being leaves, we either perform the total neighbor switch* $T:=S_{v\to u}^{B}\left(T\right)$ *or double neighbor switch* $T:=S_{v\to u,u^{+}}^{B,C}\left(T\right)$ *until the degree of u is not equal to* Δ(G).

One can observe that a double neighbor switch is needed to ensure that $\deg_{T}u$ can be increased exactly to Δ(G). Since $\deg_{T}u$ increases after each switch, only the finite number of switches is required. In case the tree *T* obtained after the first stage differs from $S_{\Delta \left(G\right)-1,n-\Delta \left(G\right)-1}$, we perform the second stage if there exist at least two vertices *w*_1_ and *w*_2_ in $N_{T}\left(u\right)$ with $\deg_{T}w_{1}\geq \deg_{T}w_{2}\geq 2$ or jump directly to Stage 3 otherwise.

Stage 2: *For each distinct w*_1_ *and w*_2_ *in* $N_{T}\left(u\right)$ *with* $\deg_{T}w_{1}\geq \deg_{T}w_{2}\geq 2$ *perform a total neighbor switch* $T:=S_{w_{2}\to w_{1}}^{B}\left(T\right)$.

After each iteration, the number of vertices in $N_{T}\left(u\right)$ with degree at least two decreases by one. Thus, Stage 2 terminates after a finite number of switches leaving at most one vertex $w\in N_{T}\left(u\right)$ with degree at least two. Finally if *T* still differs from $S_{\Delta \left(G\right)-1,n-\Delta \left(G\right)-1}$, the third stage is required.

Stage 3: *While there exists vertex v in* $N_{T}\left(w\right)\setminus \left\{u\right\}$ *with* $\deg_{T}v\geq 2$ *perform a total neighbor switch* $T:=S_{v\to w}^{B}\left(T\right)$.

Since the number of neighbors of *w* with degrees at least two decreases after each switch, Stage 3 terminates after finite number of steps with all neighbors of w, except for *u*, being leaves, that is, $T=S_{\Delta \left(G\right)-1,n-\Delta \left(G\right)-1}$. Note that each iteration of Stages 1–3 produces a tree with a higher *s*-metric due to Lemmas 2, 6 and Corollary 3.

## 3. Hardness Results

In this section, we study the computational complexity of both the *m*-SF and the *s*-SF Spanning Tree problem. The following known fact is used:

**Theorem 8** (Kleitman and West, 1991). *Any connected graph of order n with minimum vertex degree at least* 3 *has a spanning tree with at least* n∕4+2 *leaves.*

We start by investigating the complexity of our problems for cubic graphs.

**Theorem 9.** *The m*-SF Spanning Tree *problem is* APX*-hard for cubic graphs.*

*Proof.* Let *G* be a cubic graph on *n* vertices and *T* be a spanning tree with ℓ=ℓ(T) leaves and $n_{i}=n_{i}\left(T\right)$ vertices of degree *i*, i∈{2,3}. Then

(10) $$m\left(T\right)=\ell +4n_{2}+9n_{3},$$

with the numbers *n_i_* satisfying the equalities $\ell +n_{2}+n_{3}=n$ and $\ell +2n_{2}+3n_{3}=2\left(n-1\right).$ Deriving *n*_2_ and *n*_3_ from these equalities gives us

(11) $$n_{2}=n+2-2\ell ,n_{3}=\ell -2.$$

After substituting these expressions into Equation (10), we get

(12) m(T)=2ℓ+4n−10.

Thus, finding a spanning tree with maximum *m*-metric in this case is polynomially equivalent to finding a spanning tree with maximum number of leaves (MaxLeaf problem). For cubic graphs, the latter problem was shown to be APX-hard by Bonsma (2012). Thus, we prove the APX-hardness of the *m*-SF Spanning Tree problem by providing an L-reduction (Papadimitriou and Yannakakis, 1991) from MaxLeaf.

Given an optimization problem *P* and an instance *I* of this problem, we use $opt_{P}\left(I\right)$ to denote the optimum value of *I*, and $val_{P}\left(I,S\right)$ to denote the value of a feasible solution *S* of instance *I*. Let *A* and *B* be two optimization problems. The problem *A* is said to be L-reducible to *B* if there exist polynomial-time computable functions *f*, *g* and constants α,β>0 such that

(L1) *f* maps any instance *I* of *A* to an instance f(I) of *B* such that $opt_{B}\left(f\left(I\right)\right)\leq \alpha \cdot opt_{A}\left(I\right)$;

(L2) for any instance *I* of *A* and a solution S′ of the instance f(I), *g* maps S′ to a solution *S* for *I* such that $\left|val_{A}\left(I,S\right)-opt_{A}\left(I\right)|\leq \beta \cdot |val_{B}\left(f\left(I\right),S\prime \right)-opt_{B}\left(f\left(I\right)\right)\right|$.

Let $T^{mopt}$ be an *m*-optimal spanning tree of *G* and $\ell^{*}$ be the maximum number of leaves in spanning trees of *G*. Note that $\ell^{*}\geq n∕4+2$ by Theorem 8, and therefore, $n\leq 4\ell^{*}-8$. Then using Equation (12) we get

$$\tau_{1}\left(G\right)=m\left(T^{mopt}\right)=2\ell \left(T^{mopt}\right)+4n-10\leq 2\ell^{*}+16\ell^{*}-32\leq 18\ell^{*}.$$

Moreover, for every spanning tree *T* of *G* we have $\frac{1}{2}|m\left(T\right)-m\left(T^{mopt}\right)|=|\ell \left(T\right)-\ell^{*}|$. As a result, Equation (12) implies an L-reduction with identity mappings *f* and *g* and constants α=18 and $\beta =\frac{1}{2}$, thus proving the theorem.

**Theorem 10.** *The s*-SF Spanning Tree *problem is* NP*-hard for cubic graphs.*

*Proof.* For the reduction, we use the following problem proved to be NP-complete by Lemke (1988):

*Instance:* A connected cubic graph *G* of order *n*.

*Question:* Is there a spanning tree of *G* without vertices of degree 2?

According to Equation (11), $n_{2}=n_{2}\left(T\right)=n+2-2\ell \left(T\right)$. Thus, the answer for the problem's question is negative if *n* is odd. Hence, we concentrate only on the case when n≥4 is even, thus *n*_2_ is even as well. We show that among all trees *T* of order *n* with Δ(T)≤3, the trees without vertices of degree 2 have the highest *s*-metric. Indeed, the following claim holds:

**Claim 11.** *If* Δ(T)≤3 *and* n≥4 *are even, then* s(T)≤6n−15*. The equality holds if and only if T has no vertices of degree* 2.

*Proof.* If *T* has no vertices of degree 2, then Equation (11) implies $\ell =\ell \left(T\right)=\frac{n+2}{2}$. Furthermore, $s\left(T\right)=3m_{1}+9m_{3}$, where *m*_1_ is the number of edges incident to a leaf and *m*_3_ is the number of edges with both ends of degree 3. Obviously, $m_{1}=\ell$ and $m_{3}=n-1-\ell$, thus yielding s(T)=6n−15.

Now suppose that *T* has $n_{2}\geq 2$ vertices of degree 2. Let *u* and *v* be two vertices of degree 2 lying on a path $P_{T}\left(u,v\right)$ and $\deg_{T}u^{+}\geq \deg_{T}v^{-}$. Iteratively applying a total neighbor switch $S_{v\to u}^{B}$ for all pairs of vertices *u* and *v* of degree 2, we obtain a tree with higher *s*-metric (due to Corollary 3) and without vertices of degree 2. This proves the claim. □

Thus, $\tau_{2}\left(G\right)=6n-15$ if and only if *G* has a spanning tree without vertices of degree 2. This concludes the proof.

Next, we consider bipartite graphs.

**Theorem 12.** *The m*-SF Spanning Tree *and s*-SF Spanning Tree *problems are* NP*-hard for bipartite graphs.*

*Proof.* We present a polynomial-time reduction from the NP-complete 3-Dimensional Matching (3-DM) problem (Garey and Johnson, 1979):

*Instance*: Pairwise disjoint sets *X*, *Y*, *Z* of cardinality *n*, and a collection ℳ of *m* three-element sets, where each M∈ℳ includes exactly one element from each of *X*, *Y*, and *Z*.

*Question*: Is there a set of pairwise disjoint members of ℳ (a *perfect* 3*-dimensional matching*), whose union is X∪Y∪Z?

**FIG. 2.**
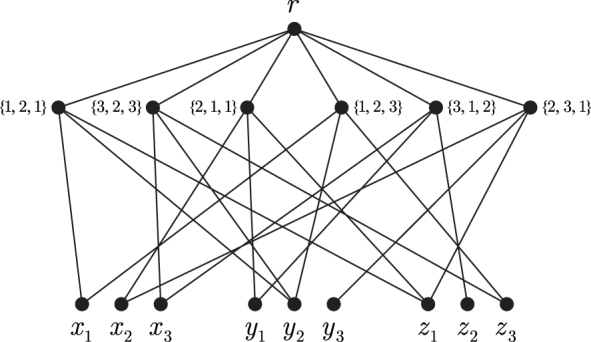
An example of the graph *G* for n=3, $X=\left\{x_{1},x_{2},x_{3}\right\}$, $Y=\left\{y_{1},y_{2},y_{3}\right\}$, $Z=\left\{z_{1},z_{2},z_{3}\right\}$, and $ℳ=\left\{\{x_{1},y_{2},z_{1}\right\},\left\{x_{3},y_{2},z_{3}\right\},\left\{x_{2},y_{1},z_{1}\right\},$ $\left\{x_{1},y_{2},z_{3}\},\left\{x_{3},y_{1},z_{2}\right\},\left\{x_{2},y_{3},z_{1}\right\}\right\}$. Here each vertex labeled {p,q,r} represents a set $\left\{x_{p},y_{q},z_{r}\right\}$.

Let Q=(X,Y,Z,ℳ) be an instance of 3-DM. We construct a graph $G=G_{Q}$ on 3n+m+1 vertices as follows. The vertex set of *G* is the disjoint union {r}∪A∪B, where A=ℳ, B=X∪Y∪Z, and *r* is the special root vertex. The edge set includes all edges *ra*, a∈A, as well as the edges *Mx*, *My*, and *Mz* for each M={x,y,z}∈A (Fig. 2). We may assume that *G* is connected. Note also that *G* is a bipartite graph with the parts *A* and {r}∪B.

For a vertex *v* of *G* and a subset W⊆V(G) let us denote by (v:W) the set of edges connecting *v* to vertices in *W*.

**Lemma 13.** *There are spanning trees T*_1_ *and T*_2_ *in G, both containing all edges of* (r:A)*, with* $m\left(T_{1}\right)=\tau_{1}\left(G\right)$ *and* $s\left(T_{2}\right)=\tau_{2}\left(G\right)$.

*Proof.* We provide the proof for the *s*-metric, the proof for the *m*-metric is similar. Among the optimal spanning trees of *G*, let *T*_2_ be the one with the maximum number of edges from (r:A). We claim that *T*_2_ contains all these edges.

Suppose for a contradiction that the set C⊆A of all vertices that are adjacent to *r* in *T*_2_ differs from *A*. Then there must be a vertex b∈B with $P_{T_{2}}\left(r,b\right)$ having two edges, such that set $D=N_{T_{2}}\left(b\right)\cap \left\{A\setminus C\right\}$ is nonempty. By Lemma 5, since $\deg_{T_{2}}r^{+}=\deg_{T_{2}}b^{-}$ and $\deg_{T_{2}}r\geq 1$, we can apply total neighbor switch $S_{b\to r}^{D}$ to construct a spanning tree $T\prime_{2}$ from *T*_2_ with $s\left(T\prime_{2}\right)\geq s\left(T_{2}\right)$, and the root *r* having more neighbors in $T\prime_{2}$ than it has in *T*_2_.

Any spanning tree *T* of *G* containing all edges of (r:A) has m+3n edges, 3n(m−1) paths of length three (each of the 3n edges of the tree connecting *A* and *B* induces exactly m−1 such paths), and m(m−1)∕2+3n paths of length two that are not formed by a pair of edges between *A* and *B*. There are $3\delta_{4}+\delta_{3}$ remaining paths of length two, where $\delta_{i}$ is the number of vertices in *A* that have degree *i* in the tree. Indeed, a vertex v∈A with j∈{0,1,2,3} neighbors from *B* in the tree contributes no such path in case of j∈{0,1}, one such path in case of j=2, and three such paths in case of j=3. Thus by Proposition 1

$$m\left(T\right)=m^{2}+m+12n+6\delta_{4}+2\delta_{3},s\left(T\right)=m^{2}+3mn+6n+6\delta_{4}+2\delta_{3}.$$

Since |B|=3n, we have $3\delta_{4}+2\delta_{3}\leq 3n$ and $6\delta_{4}+2\delta_{3}\leq 6\delta_{4}+4\delta_{3}\leq 6n$. Hence, $6\delta_{4}+2\delta_{3}\leq 6n$ with equality holding if and only if $\delta_{3}=0$ and $\delta_{4}=n$.

A perfect 3-DM ${ℳ}^{*}=\left\{M_{1},\ldots ,M_{n}\right\}$ induces the spanning tree $T_{{ℳ}^{*}}$ that contains all edges from (r:A) and edges ax,ay,az for each $a=\left\{x,y,z\right\}\in {ℳ}^{*}$. For this tree we have $\delta_{4}=n$ and


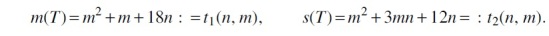


Conversely, every spanning tree *T* that contains all edges from (r:A) and $m\left(T\right)=t_{1}\left(n,m\right)$ or $s\left(T\right)=t_{2}\left(n,m\right)$ (and thus $\delta_{4}=n$) arises from a perfect 3-DM.

By Lemma 13, the graph *G* satisfies $\tau_{1}\left(G\right)\geq t_{1}\left(n,m\right)$ (resp., $\tau_{2}\left(G\right)\geq t_{2}\left(n,m\right)$) if and only if there is a spanning tree *T* of *G* that contains all edges from (r:A) and whose *m*-metric (resp., *s*-metric) is equal to $t_{1}\left(n,m\right)$ (resp., $t_{2}\left(n,m)\right)$. The latter is true if and only if *Q* has a perfect 3-DM. □

## 4. ILP Formulations

Here we describe two ILP models for the *s*-SF Spanning Tree problem (for the *m*-SF Spanning Tree problem the approach is similar). For a given spanning tree *T* of a graph G=(V,E) of order *n*, consider the indicator variables $(x_{e})e\in E$:

(13) $$x_{e}=\left\{\begin{array}{c} \begin{array}{c} 1,e\in E\left(T\right);\\ 0,otherwise. \end{array} \end{array}\right.$$

Using Proposition 1, we can represent s(T) as

(14) $$s\left(T\right)=\sum_{\left\{e_{i},e_{j},e_{k}\right\}\in \Gamma_{3}\left(G\right)}x_{e_{i}}x_{e_{j}}x_{e_{k}}+2\sum_{\left\{e_{i},e_{j}\right\}\in \Gamma_{2}\left(G\right)}x_{e_{i}}x_{e_{j}}+\sum_{e\in E\left(G\right)}x_{e},$$

where $\Gamma_{i}\left(G\right)$ denotes the set of all paths of length *i* in *G*. To linearize (14), we introduce Boolean variables $y_{ijk}$ and $y_{ij}$ and the following constraints:

(15) $$\begin{array}{c} y_{ijk}\leq x_{e_{i}},y_{ij}\leq x_{e_{i}},\\ y_{ijk}\leq x_{e_{j}},y_{ij}\leq x_{e_{j}},\\ y_{ijk}\leq x_{e_{k}},y_{ij}\geq x_{e_{i}}+x_{e_{j}}-1,\\ y_{ijk}\geq x_{e_{i}}+x_{e_{j}}+x_{e_{k}}-2, \end{array}$$

for every $\left\{e_{i},e_{j},e_{k}\right\}\in \Gamma_{3}\left(G\right)$ and $\left\{e_{i},e_{j}\right\}\in \Gamma_{2}\left(G\right)$, which are equivalent to $y_{ijk}=x_{e_{i}}x_{e_{j}}x_{e_{k}}$ and $y_{ij}=x_{e_{i}}x_{e_{j}}$. Thus the objective function (14) can be rewritten as

(16) $$s\left(T\right)=\sum_{\left\{e_{i},e_{j},e_{k}\right\}\in \Gamma_{3}\left(G\right)}y_{ijk}+2\sum_{\left\{e_{i},e_{j}\right\}\in \Gamma_{2}\left(G\right)}y_{ij}+\sum_{e\in E\left(G\right)}x_{e}.$$

We use two types of constraints to describe the spanning trees. The first type is the extended formulation of Martin (1991), which uses auxiliary variables

(17) $$z_{\left(v,w\right)}^{r},z_{\left(w,v\right)}^{r}\geq 0foreveryr\in V\left(G\right),vw\in E\left(G\right),$$

where $z_{\left(v,r\right)}^{r}=0$ for every r∈V(G) and vr∈E(G). A 0/1-vector *x* describes a spanning tree of *G* if and only if these variables satisfy the constraints

(18) $$\begin{array}{cc} x_{vw}-z_{\left(v,w\right)}^{r}-z_{\left(w,v\right)}^{r} & =0,r\in V\left(G\right),vw\in E\left(G\right),\\ \sum_{vw\in E\left(G\right)}z_{\left(v,w\right)}^{r}=1,r,w\in V\left(G\right),r\neq w,\\ \sum_{vr\in E\left(G\right)}z_{\left(v,r\right)}^{r}=0,r\in V\left(G\right). \end{array}$$

The second type exploits the Miller–Tucker–Zemlin (MTZ) constraints (Miller et al., 1960). We introduce the auxiliary variables

(19) $$\begin{array}{c} z_{\left(v,w\right)},z_{\left(w,v\right)}\in \left\{0,1\right\}foreveryvw\in E\left(G\right),\\ t_{v}\in \left[0,n-1\right]foreveryv\in V\left(G\right), \end{array}$$

and constraints

(20) $$\begin{array}{c} x_{vw}-z_{\left(v,w\right)}-z_{\left(w,v\right)}=0,vw\in E\left(G\right),\\ \sum_{vw\in E\left(G\right)}z_{\left(v,w\right)}=1,w\in V\left(G\right)\setminus \left\{r\right\},\\ \sum_{vr\in E\left(G\right)}z_{\left(v,r\right)}=0,\\ t_{v}-t_{w}+nz_{\left(v,w\right)}\leq n-1,v,w\in V\left(G\right),vw\in E\left(G\right), \end{array}$$

where r∈V(G) is some fixed vertex. Finally we add the additional constraint

(21) $$s\left(T\right)=\sum_{\left\{e_{i},e_{j},e_{k}\right\}\in \Gamma_{3}\left(G\right)}y_{ijk}+2\sum_{\left\{e_{i},e_{j}\right\}\in \Gamma_{2}\left(G\right)}y_{ij}+\sum_{e\in E\left(G\right)}x_{e}\leq n\left(n-\Delta \left(G\right)-1\right)+\Delta^{2}\left(G\right),$$

defined by Theorem 7, which turns out to significantly improve the algorithm running times. Maximization of the objective (16) subject to the constraints (15), (18), (21) is further referred to as Martin formulation, while maximization of Equation (16) subject to Equations (15), (20), (21) as MTZ formulation.

## 5. Experimental Results

In this section, we investigate the practical aspects of scale-free spanning tree problems by conducting computational experiments for various simulated and experimental data sets to evaluate the performance of the ILP models. All computations below were performed on a standard laptop with 2.0 GHz dual core processor and 16 GB of RAM, and ILP problems were solved using Gurobi 8.1.

### 5.1. Synthetic data

#### 5.1.1. Synthetic graphs

We used graphs from the following synthetic data sets:

*Erdős-Rényi graphs* constructed by adding each possible edge uniformly and independently with the probability p=4.25∕n. The number of nodes *n* in our experiments varied from 10 to 40 (corresponding to the sizes of HCV outbreaks analyzed later).

n×m *grid graphs* (Cartesian products of paths *P_n_* and *P_m_*) with n,m=4,…,7.

*Scale-free graphs* of two types generated using NetworkX library (Hagberg et al., 2008): those based on the classical Barabási and Albert (1999) model and those constructed with NetworkX default parameters. The latter graphs are usually denser.

**FIG. 3.**
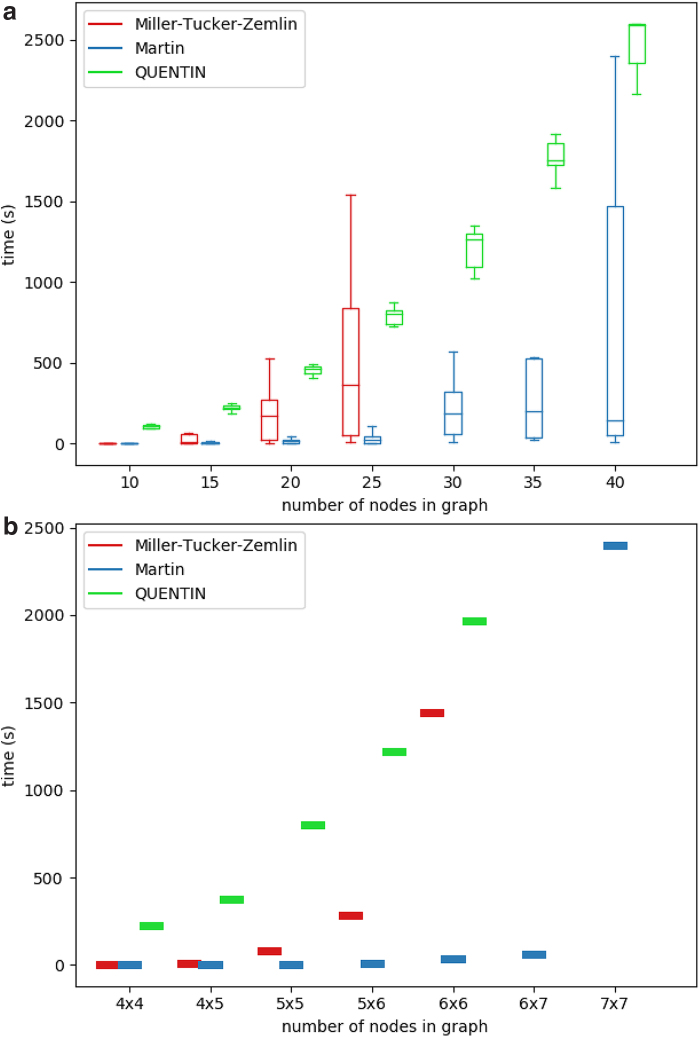
Running times of ILP solver and QUENTIN on Erdős–Rényi graphs **(a)** and grids **(b).** ILP, integer linear programming.

**FIG. 4.**
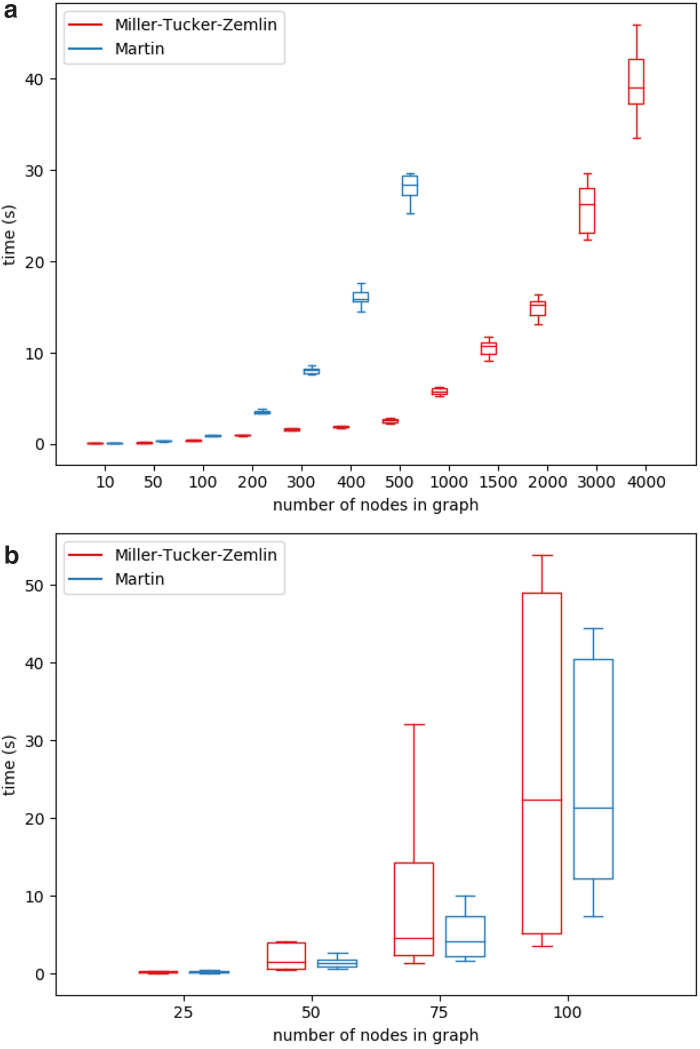
Running times of ILP solver on Barabási–Albert **(a)** and NetworkX **(b)** scale-free graphs.

For all synthetic data sets except for grid graphs, we generated 10 graphs per node number. Figures 3 and 4 illustrate the running times of the ILP solver on both the MTZ formulation and the Martin formulation compared with the published tool QUENTIN (Skums et al., 2018) runtimes for all four simulated graph classes.^1^ The results demonstrate that for those graph classes, the ILP algorithms in average perform much better than in the worst case and are able to produce optimal results in a reasonable amount of time. Moreover, for considered graph sizes, they outperform QUENTIN. For Erdős–Rényi graphs and grids (Fig. 3), which are characterized by relatively large sets of feasible solutions, the Martin formulation was superior to MTZ and QUENTIN, while for Barabási–Albert scale-free graphs (Fig. 4a), the MTZ formulation was leading to the faster algorithm. In general, the ILP approach allows to solve the problem within minutes or few hours for small-to-medium-sized problems (up to several dozens of vertices) on Erdős–Rényi graphs and grids, and for medium-sized problems (several hundred vertices) on scale-free graphs.

#### 5.1.2. Simulated outbreaks

We simulated outbreaks over scale-free Barabási–Albert contact networks of n=10−30 nodes using the following model. The infection spreads over each network according to the susceptible infected (SI) model (Newman, 2010) with the transmission rate $\rho =10^{-2}$. Each infected individual is assumed to carry a viral sequence of length m=13200, and at each transmission event, the source's sequence is transmitted to the recipient. Sequence evolution is described by a skyline model with the piecewise constant decreasing mutation rate, that is, viral sequences mutate at the basic rate of $\mu =10^{-5}$ changes/position/time unit, and the mutation rate is decreasing by 30% every τ=100 time units. This model captures the decrease of the speed of intrahost evolution as the infection progresses from an acute to a persistent stage (De Maio et al., 2016; Icer et al., 2020).

For each simulated outbreak, we compared the performance of the ILP algorithm for the Martin formulation, with the standard approach based on the phylogenetic trait inference (Sagulenko et al., 2018). First, we constructed a maximum likelihood phylogeny using MEGA (Kumar et al., 2018). Each patient was encoded by a discrete trait, and the marginal likelihood ancestral traits were reconstructed using the Felsenstein pruning algorithm (Felsenstein, 2004) with the pairwise between-trait transition rates equal to ρ. Inferred transmission links then correspond to trait changes along the phylogeny branches. The genetic relatedness network *G_R_* used as an input for the ILP was constructed using a threshold-based approach suggested by Kosakovsky Pond et al. (2018). A pair of vertices of *G_R_* are adjacent, if the Hamming distance between the corresponding sequences does not exceed a threshold *t* that was estimated as the minimal integer such that the graph *G_R_* is connected. The obtained graph was further sparsed out by applying the same procedure to each of its biconnected component.

**FIG. 5.**
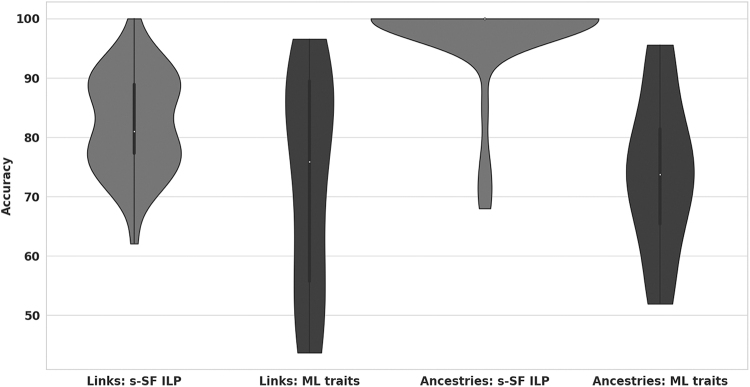
Accuracy of *s*-SF ILP model compared with the phylogenetic trait inference algorithm.

The results of algorithms' comparison are shown in Figure 5. We measured algorithm accuracy by the proportion of correctly inferred transmission links and transmission ancestries (i.e., pairs, ancestor/descendant). *s*-SF-based ILP clearly outperformed the phylogenetic approach: the average transmission link detection accuracy was 82.44% for the former and 72.61% for the latter, while the average transmission ancestry detection accuracies were 97.48% and 73.96%, respectively.

### 5.2. Data from hepatitis C outbreaks

We applied the concept of scale-free spanning trees to the graphs arising from the benchmark data set consisting of several epidemiologically curated HCV outbreaks investigated by the CDC (Campo et al., 2016; Glebova et al., 2017; Skums et al., 2018). This data set comprises HCV quasispecies populations sampled from 81 infected individuals involved in 10 viral outbreaks. Each population consists of RNA sequences of HCV hypervariable region 1 (HVR1) of length 264 bp. Transmission histories of the outbreaks (“who infected whom”) are known as a result of epidemiological investigations. In this case, we are dealing with intrahost viral populations rather than single sequences, and therefore, we compared the proposed approach with QUENTIN, which has been specifically designed to handle such data (Skums et al., 2018).

For each outbreak, the genetic relatedness network *G_R_* was constructed using the threshold-based approach suggested by Campo et al. (2016). The vertices of *G_R_* are adjacent, if the *minimal* Hamming distance between the sets of sequences sampled from these patients does not exceed the threshold *t*. The threshold value was estimated as described in Subsection 5.1.2. Next, the ILP algorithm for the Martin formulation has been applied to *G_R_*. For all outbreaks, the ILP problem has been solved to optimality.

We tested the accuracy of inference of transmission links and identification of the superspreaders (the sources of majority of infections). The results are reported in Table 1. The superspreaders correspond to vertices of highest degrees in *s*-optimal and *m*-optimal trees for 9 out of 10 outbreaks. It should be noted that all algorithms incorrectly identified a superspreader for the same outbreak. It is the only outbreak where the virus was transmitted via a nonsocial interaction (namely, through blood transfusions), while all other outbreaks were associated with unsafe injection practices or sexual contacts. For those outbreaks, both ILP approaches correctly recovered 92% of transmission links and all ancestor/descendant pairs, thus outperforming QUENTIN.

**Table 1.** Results on Experimental Data with Different Models

Methods	Evaluation metric		
	(A)	(B)	(C)
QUENTIN	0.9	0.78	0.98
*s*-SF	0.9	0.92	1.0
*m*-SF	0.9	0.92	1.0

(A) Superspreader inference accuracy, (B) accuracy of transmission link inference, and (C) accuracy of transmission ancestry inference.

## 6. Discussion

In genomic epidemiology, reconstruction of viral transmission histories from genomic data is fundamental for the investigation of outbreaks and understanding of epidemic spread. Genomic analysis has become one of the major tools for the investigation of outbreaks and surveillance of transmission dynamics (Armstrong et al., 2019; Knyazev et al., 2020). Naturally, graphs are the primary models used in such studies (Wertheim et al., 2014; Campo et al., 2016; Ragonnet-Cronin et al., 2019). In many settings, graph-based methods have been shown to be more efficient to ascertain transmission links compared with methods based on binary phylogenies (Wertheim et al., 2014), as phylogenetic clades are not easily resolvable into transmission clusters and pairs (Lewis et al., 2008; Hughes et al., 2009; Kouyos et al., 2010), while the statistical support for a clade does not necessarily indicate the statistical support for a relationship between individual genomes inside a clade (Volz et al., 2012; Wertheim et al., 2014). However, in many cases, transmission links cannot be inferred using the genomic data alone (Jombart et al., 2014; Villandre et al., 2016). It leads to the need to introduce additional constraints on the reconstructed transmission networks or utilize more complicated objectives.

As a result, the associated algorithmic problems become harder. In this article, we studied one such problem—scale-free spanning tree problem—that arises in epidemiological studies of viruses whose spread is highly influenced by social networks of contacts between susceptible individuals. This includes HIV, HCV, and other pathogens transmitted through sexual contact or needle sharing. We demonstrated that this problem in its two possible algorithmic formulations is NP-hard, even if restricted to relatively simple graph classes. However, it admits an ILP formulation allowing to efficiently solve the problem for small-to-medium networks. It is often enough for the vast majority of outbreaks of HIV and HCV that involve dozens of infected individuals.

However, some outbreaks involve hundreds or even thousands of hosts, and in such cases, more scalable algorithmic solutions are needed. Thus, an important open problem is to establish whether constant or logarithmic approximation exists for the *m*-SF Spanning Tree and *s*-SF Spanning Tree problems. In this context, it would be interesting to explore the relationships between scale-free spanning tree problems and max-leaf spanning tree problems. The latter is a well-studied combinatorial problem (Griggs et al., 1989; Galbiati et al., 1994), which seems to be the closest to our problem. Indeed, both problems aim to find a “star-like” spanning tree; furthermore, several reduction schemes for the proof of NP-completeness used by us exploit this relationship. Importantly, Lu and Ravi (1998) and Reich (2016) showed that the max-leaf spanning tree problem is approximable within a constant factor. Although the problems are far from being equivalent, it may seem reasonable for future studies to try to adopt algorithmic machinery developed for the max-leaf spanning tree problem to the scale-free spanning tree problem.

## Notes

^1^
Running times for MTZ formulation on grids and Martin formulation on Barabási–Albert scale-free graphs are plotted only for smaller *n*, since for large values they are significantly higher than for the other formulation. In particular, Martin formulation on Barabási–Albert scale-free graphs works ∼ 150 seconds for 1000 vertices, ∼ 480 seconds for 1500 vertices, and exceeds 1800 seconds for 2000 and more vertices. QUENTIN running times are not plotted in Figure 4, since they already exceed timeout of 3600 seconds for 50 vertices for both scale-free graphs.
